# Identifying Who Improves or Maintains Their Food Literacy Behaviours after Completing an Adult Program

**DOI:** 10.3390/ijerph17124462

**Published:** 2020-06-21

**Authors:** Andrea Begley, Ellen Paynter, Lucy Butcher, Vanessa Bobongie, Satvinder S. Dhaliwal

**Affiliations:** 1 School of Public Health, Curtin University, Perth 6102, Australia; ellen.paynter@curtin.edu.au (E.P.); s.dhaliwal@curtin.edu.au (S.S.D.); 2 Foodbank Western Australia, Perth Airport 6105, Australia; lucy.butcher@foodbankwa.org.au (L.B.); vanessa.bobongie@health.wa.gov.au (V.B.); 3 Duke-NUS Medical School, National University of Singapore, 8 College Rd, Singapore 169857, Singapore

**Keywords:** food literacy, intervention, diet, behaviour change, outcome, evaluation

## Abstract

Food Sensations for Adults is a free four-week nutrition and cooking program that teaches low- to middle-income individuals food literacy. This research aimed to compare demographic characteristics of participants who completed the program’s follow-up questionnaire three months after program completion and assess whether food literacy and dietary behaviour changes were improved or maintained. Statistical analysis methods used factor scores of the plan and manage, selection, and preparation domains to examine mean self-reported changes in food literacy. Tertile stratification methods calculated changes in participants who had low, middle, and high end-of-program food literacy scores, and multivariable regression analysis explored the associations. The follow-up results (*n* = 621) demonstrated a statistically significant factor score increase in plan and manage (3%) and selection (7.2%) domain scores, and a decrease in the preparation score (3.1%), and serves of consumed vegetables (7.9%), but were still significantly higher than at the start of the program. At follow-up, participants with low food literacy at the program end significantly improved their follow-up domain scores for plan and manage (60%) and selection (73.3%), and participants with moderate or high food literacy at the program end maintained their follow-up scores. A food literacy program can support adults to improve and maintain their food literacy behaviours and maintain dietary behaviour change; therefore, strategies to support this continued change must be considered.

## 1. Introduction

Food literacy is ‘composed of a collection of inter-related knowledge, skills, and behaviours required to plan, manage, select, prepare, and eat food to meet needs and determine intake’ [1]. The four domains—planning and management, selection, preparation, and cooking and eating—are essential components of a food literate person. Food literacy is a relatively new term that incorporates the strategies used in many nutrition education programs and more specifically, cooking skill interventions to improve dietary behaviours and enhance health outcomes [2,3]. These programs are conducted in community settings and target a wide range of groups, including vulnerable populations.

Best practices for providing low-income audiences with effective nutrition education indicate that conducting outcome (follow-up) evaluation and assessing maintained behaviour change are two critical evaluation practices [4]. A current challenge for program evaluation is developing tools and evaluation methods that measure food literacy and differentiate participants based on behaviours [5,6]. Despite best practice recommendations and evaluation challenges, food literacy programs generally do not report on outcomes or include follow-up evaluations [7]. Programs that use food literacy strategies that do investigate long-term outcomes vary in their follow-up period between one to six months [7,8,9,10,11,12]; however, time frames have also been as short as one week [13] and as long as two years [14]. Programs conducted in ‘real world’ community settings do not generally attain high follow-up response rates [2] due to the voluntary nature of participation and the frequently low English literacy of attendees. For example, the United Kingdom’s (UK) ‘Eat Better Feel Better’ cooking program recorded that 53% of participants completed the pre- and post-program questionnaire, but less than 15% completed the three-month follow-up questionnaire [11]. Not only this, but the United States’ ‘Cooking Matters’ [15] and the UK’s ‘Jamie’s Ministry of Food’ [7] reported that less than 40% of participants completed the six-month follow-up evaluation. Regardless of a program’s success rate in retaining follow-up respondents, long-term maintenance of improved eating patterns and food literacy behaviours were reported [7,11,15].

There are only two Australian food literacy programs that have published follow-up evaluation findings [8,9,10]. The most recent of these programs, Jamie Oliver’s ‘Ministry of Food’ Australian version, found that a participant’s improvements in vegetable consumption and cooking confidence remained evident six months after the program ended. However, despite monetary incentives, only 14% of participants completed the follow-up evaluation [10]. Since 1992, the Western Australian (WA) Department of Health has funded a state-wide food literacy program. The 2011–2014 Food Cent$ Project evaluation found that 12% of the total intervention sample completed a follow-up evaluation six weeks after the project’s completion; however, interpreting the results is difficult because the intervention delivery varied in length from one to eight sessions. Nevertheless, improvements in knowledge and fruit consumption in low socio-economic participants were found at follow-up [9]. The results from the earlier 1992 Food Cent$ Project demonstrated a self-reported reduction in discretionary food intake by 36% of attendees who completed the six-week follow-up [8]. However, there is currently no existing literature that provides a nuanced view regarding who the program significantly affects and to what extent changes are maintained, improved or decreased at follow-up.

Since 2007, Foodbank WA has delivered a food literacy program across WA [16], with the Food Sensations for Adults (FSA) program introduced in 2011. The program was extensively redeveloped in 2015 by Foodbank WA to align with the Australian food literacy model [1] and the best practice criteria [17] and has been funded by the WA Department of Health since 2016. FSA is a free program designed for individuals from low- and middle-income households who want to improve their food literacy. It includes four 2.5-h sessions (10 contact hours total), but an online video conference delivery method is utilised to increase accessibility across WA. The sessions include nutrition education and cooking workshops designed to support self-efficacy and motivation and discuss a range of topics such as the Australian Dietary Guidelines [18], label reading, meal planning, and budgeting skills. Continuity is maintained throughout all the programs—the first three sessions cover comprehensive food literacy content but the final session meets the needs of the audience by including a tailored nutrition session [19], in which participants can select one topic that they would like to discuss (e.g., mindful eating or healthy lunchboxes, snacks, or gardening,). The post-program evaluation demonstrated that the program improves food literacy and dietary behaviours, particularly in individuals that had low food literacy at the beginning of the program [19,20].

This research aims to determine whether participants continue to improve or maintain food literacy behaviours and selected dietary practices three months after the program’s end. The program’s long-term influence is established by assessing participants’ food literacy and dietary behaviours between the end-of-program and follow-up questionnaires, and analysing factors that associate with continuing improvements or maintained improvements in food literacy behaviours between the beginning and end of the program.

## 2. Materials and Methods 

### 2.1. Study Design

Cross-sectional questionnaires were conducted on three occasions—the program’s beginning and end, and three months after completion. Participants completed hard copy questionnaires at the beginning and end of the program and non-attendees completed the questionnaire online. The end-of-program questionnaire asked participants to indicate how they would like to complete their voluntary follow-up questionnaire: online, posted hard copy, or telephone. The participants were not directly paid for completing the follow-up questionnaire; rather, they were automatically entered into a draw every two months to win a A$200 supermarket voucher. Not all programs were evaluated due to literacy levels in ethnically diverse groups or confidentiality issues such as prison groups and not all participants consented to the evaluation. During the time period for this study, 2,841 participants attended one or more FSA sessions.

### 2.2. Questionnaire

Within the first questionnaire, participants completed a 14-item food literacy behaviour checklist and answered questions regarding their food literacy-related practices, selected dietary behaviours, and socio-demographic information. The end-of-program questionnaire included the same 14-item food literacy behaviour checklist and questions regarding selected dietary behaviours, and the follow-up questionnaire included the same 14-item food literacy behaviour checklist and questions regarding food literacy-related practices, selected dietary behaviours with an additional question on the barriers to change experienced.

Within each of the questionnaires, different sets of questions were asked. The first included questions regarding eight socio-demographic characteristics: sex, age group, household composition, education level, employment status, socio-economic index, whether they were born in Australia and whether they were Aboriginal or Torres Strait Islanders. All questionnaires included two questions regarding self-reported dietary behaviours—self-reported intake of fruit and vegetables (serves per day) and the consumption frequency of fast food meals and sugar-sweetened drinks. Dietary behaviour questions included images of serve sizes for fruit and vegetables and examples of fast food meals and sugar-sweetened drinks from the Australian Guide to Healthy Eating to guide participants [18]. Questions were also asked regarding the perceptions concerning the cost of healthy food compared to unhealthy food. Depending on the questionnaire, additional questions regarding food literacy-related practices were also included. The first questionnaire included two questions that examined the responsibility of preparing meals and food shopping, and the first and follow-up questionnaire asked the participants to rate their cooking skills. In the follow-up questionnaire, a two-part question was included to investigate potential barriers that may have prevented change. Participants were asked whether they had experienced difficulties or encountered obstacles in establishing food-related changes at home and, if confirmed, were asked to identify these issues (refer to Supplementary Materials). Facilitators requested that participants complete all questions, but this did not always occur and the online survey did not force participants to respond to all questions, so there was missing data.

### 2.3. Data Analysis

The statistical analysis was completed using SPSS Statistics version 25 (IBM Corporation, Armonk, NY, USA). The *p*-value < 0.05 was considered statistically significant. Fruit and vegetable intake was treated as a continuous variable, and all other variables were treated as categorical.

#### 2.3.1. Demographic Characteristics

A participant’s demographic characteristics—sex, age group, household composition, education level, employment status, socio-economic index, whether they were born in Australia, and whether they were Aboriginal or Torres Strait Islanders—were used to identify participants less likely to complete the follow-up questionnaire. Chi-square tests were used to compare the demographic information of attendees who completed both the end-of-program and follow-up questionnaire against those who did not complete the follow-up questionnaire.

#### 2.3.2. Food Literacy Behaviour Factors and Tertiles

The food literacy behaviour questions were used as the foundation for three food literacy behaviour domains in each questionnaire: plan and manage, selection and preparation [21]. The factor loading score cut-off (0.4) determined that 11 of the 14 questions were included in one or more of the three domains identified by the exploratory factor analysis [21]. The response to each question was numbered, based on ‘never’ (1), ‘sometimes’ (2), ‘most of the time’ (3), or ‘always’ (4). In this study, the numbered response was multiplied by each factor loading score and summed to form the final factor score for each participant’s food literacy behaviour domain in each questionnaire.

To examine food literacy behaviour changes between the questionnaires, an innovative statistical approach was applied. The food literacy behaviour score was first allocated between three quantiles or tertiles. In clinical and epidemiological studies, quantiles are used in an analysis to separate distribution of data into groups and identify those more at risk of specific outcomes [22]; when specific values for separation are unknown, a defined variable is used to separate the distribution into three tertiles [23]. These variables can include clinical tests, biomedical scores, or dietary intake. For this study, the three literacy behaviour factor scores were used as variables to identify those who were most, moderate, and least at risk of low food literacy. The food literacy behaviour scores from the first questionnaire were used to define the tertiles at all stages of the research. Participants were designated a tertile for each food literacy behaviour domain at the program’s beginning, end, and follow-up, which were described as low, moderate, or high.

#### 2.3.3. Changes in Food Literacy Domains and Dietary Behaviours

McNemar–Bowker tests were used to investigate whether food literacy behaviour factor scores changed or remained the same between the end-of-program and follow-up questionnaires. The tests compared the tertile ranking of participants at these stages for the three food literacy behaviour domains—plan and manage, selection, and preparation. Paired t-tests were used to determine whether food literacy behaviours and dietary intake adjustments were maintained after the program’s completion. The three food literacy behaviour domains and the change in self-reported dietary intake of fruit and vegetable serves were reviewed in both the end-of-program and follow-up questionnaire scores. McNemar–Bowker tests were used to compare questionnaire scores of these two stages regarding self-reported intake of fast food meals and sugar-sweetened drinks and used to examine whether the program had improved cooking skills by comparing a participant’s self-reported cooking skills between their first and follow-up questionnaires.

#### 2.3.4. Variables Associated with Maintaining Improved Food Literacy

Multivariable regression analyses used a forced entry method to identify demographic(s), dietary behaviour(s) or food literacy-related practice(s) of participants who maintained or improved their moderate or high food literacy behaviour score between the end-of-program and follow-up questionnaires. Demographic characteristics included sex, age group, household composition, education level, employment status, socio-economic index, whether they were born in Australia and, whether they were Aboriginal or Torres Strait Islanders. Dietary behaviours included self-reported intake of fruit, vegetables, fast food meals, and sugar-sweetened drinks. Food literacy-related practices included the attitude towards the cost of healthy food, self-reported cooking skills, and the responsibility for preparing meals and purchasing food. Three food literacy behaviour domains—plan and manage, selection, and preparation—were analysed separately. Participants who scored moderately in the end-of-program questionnaire and moderate or high in the follow-up questionnaire, and those who scored high in both the end-of-program and follow-up questionnaires, were compared to all other participants (who scored low in both questionnaires or those who scored lower in the follow-up questionnaire). The results were displayed as an odds ratio, in which the likelihood of reporting a particular demographic or characteristic for participants who maintained or improved a moderate to high score compared to the reference answer (indicated by 1). An odds ratio greater than 1 with a significant *p*-value indicates an increased likelihood.

### 2.4. Ethics

Human Research Ethics Committee approval was obtained from Curtin University (RDHS-52-16). At the beginning of the program, each participant provided written consent to participate in the evaluation on an attendance sheet. If a participant consented to receive the follow-up questionnaire, they provided their contact details on their completed post-program questionnaire.

## 3. Results

### 3.1. Response Rate

Out of 1,855 participants consenting to the evaluation processes, 621 (33.4%) completed the follow-up survey and the remainder either did not consent to follow-up, did not respond when contacted at the three-month follow-up, or withdrew consent at follow-up. Of the participants who completed the follow-up, *n* = 534 (86.8%) completed all three questionnaire stages and *n* = 594 (95.6%) completed the end-of-program and follow-up questionnaires. Completion methods of the follow-up questionnaire included online (75.4%), telephone (19.2%), and postage of a hard copy (5.5%).

### 3.2. Demographic Characteristics of Follow-Up Participants

There were several distinct demographic characteristics between participants who completed the follow-up questionnaire compared to those who did not. Those who completed the follow-up questionnaire were more likely to be female (84.2% vs. 78.4%), older (17.9% vs. 11.3% [66 and over]), living as a couple with children (39.2% vs. 33.5%) or without children (21% vs. 16.5%), and have a higher education level (39.6% vs. 30.1% [certificate, diploma, or trade]) (see Table 1). Those who did not complete the follow-up questionnaire were more likely to be unemployed or unable to work (28.6% vs. 19.7%) and identified as Aboriginal or Torres Strait Islanders (8.6% vs. 3.4%).

### 3.3. Changes in Food Literacy Behaviour Domains and Dietary Behaviours

The mean scores for the plan and manage and selection behaviours increased in the follow-up questionnaire compared to the participant’s end-of-program questionnaire score (Table 2). The preparation scores regarding serves of fruit and vegetables lowered in the follow-up questionnaire but were still significantly higher than those at the beginning of the program.

Although the reported intake of fast food meals significantly increased between the end-of-program and follow-up questionnaires (*p* < 0.0001), the consumption frequency overall improved from the beginning of the program (*p* < 0.0001). There was no change in the reported intake frequency of sugar-sweetened drinks between the end-of-program and follow-up questionnaires (*p* = 0.063), or from the beginning of the program to follow-up (*p* = 1). However, the consumption frequency of fast food meals and sugar-sweetened drinks overall was low before the program—28.6% of participants had never consumed fast food, and 47.9% had never consumed sugar-sweetened drinks. Participants’ self-reported cooking skills were measured twice, with self-reported cooking skills significantly improving between the program’s first and follow-up questionnaires (see Table 3). Only one-quarter (25.8%) of participants who reported an inability to cook at the start of the program, also reported this on follow-up, and three quarters (74.2%) reported an improvement from this. Similarly, 45.9% of participants initially reporting basic cooking skills also reported an improvement in their skills. From those participants initially reporting well-developed cooking skills, 92.1% remained at that level or improved on follow-up.

### 3.4. Food Literacy Behaviour Ranking

The follow-up scores significantly improved compared to the end-of-program scores for both plan and manage and selection domain factors (see Table 4). Regarding the plan and manage domain, out of the participants whose end-of-program results scored in the moderate tertile, 91.4% scored moderate or high on follow-up, and 81.2% maintained their score in the high tertile. Crucially, 60% of participants whose end-of-program results scored low improved to moderate or high scoring on follow-up. Regarding the selection factors, more than 90% of participants in the moderate tertile maintained or improved their follow-up scores, and 82.3% of participants in the high tertile maintained their high ranking. Significantly, 73.3% of participants who scored in the low tertile improved their follow-up score. There was no statistically significant difference in preparation scores between the end-of-program and follow-up scores, which indicated that participants maintained their tertile group ranking after the end of the program (see Table 4).

### 3.5. Variables Associated with Maintaining Improved Food Literacy

Analyses were completed to characterise participants who maintained or improved a moderate or high food literacy behaviour score between the end-of-program and follow-up questionnaires. One demographic factor and one food literacy-related practice were identified (see Table 5), and no dietary behaviours were found to be significantly associated. Participants that improved or maintained their moderate or high plan and manage or selection scores were twice more likely to reside in a high socio-economic area and were also more likely to report higher self-reported cooking skills at the beginning of the program. Participants improving or maintaining a moderate or high plan and manage score were 10 times more likely to report ‘can cook a wide variety of meals’ or ‘can cook almost anything’ and 3.5 times more likely to report ‘can cook basic food’. The likelihood of the association with self-rated cooking skills for selection was less pronounced, with participants 2.8 times more likely to report ‘can cook a wide variety of meals’.

Despite no statistically significant overall change of participants moving to moderate or high tertile groups in the preparation domain, participants who did maintained or improved a moderate or high score associated with self-rated cooking skills. The participants that maintained or improved their moderate or high preparation score were 3.8 times more likely to report ‘can cook almost anything’ and 2.8 times more likely to report ‘can cook a wide variety of meals’.

### 3.6. Barriers to Changes at Follow-Up

Participants were asked to report on any barriers or challenges they had experienced in the three-month follow-up questionnaire. Of those who answered the question, ‘Have you experienced any difficulties or obstacles making food-related changes at home?’ (*n* = 597), 33.3% reported difficulties or obstacles in establishing food-related changes at home, 25.9% reported taste preferences of household members, and 23.2% reported busy lifestyles to be barriers. Other barriers that participants experienced also included not wanting to change the intake of preferred foods (14.2%) and the price of healthy foods (13.2%).

## 4. Discussion

This study used an innovative statistical approach to provide insight into the long-term effects of food literacy programs, as many programs do not report on their outcome or follow-up effects. It is crucial to identify the aspects of a program that resonate with participants to assist program developers in creating strategies to support using food literacy behaviours and support healthy eating after a program’s completion. The research results are an important contribution to the understanding of food literacy program effectiveness.

The response rate for participants completing the follow-up FSA questionnaire was similar [8] and higher [25] than other comparable Australian studies. The demographic characteristic differences reported are similar to published research, which found differences in participants who completed follow-up surveys after attending food literacy or cooking skill interventions may have different characteristics. ‘Cooking Matters’ researchers found that participants that were younger and had a lower education level were least likely to complete the follow-up [15]. ‘Eat Better Feel Better’ researchers statistically compared those in the follow-up group to the original and found significant differences between the age and number of household members between the two groups—participants that were younger and had fewer household members were least likely to complete the follow-up [11]. However, other programs such as ‘Ministry of Food’ found similar participant characteristics in those who only completed the beginning and end-of-program questionnaires versus those that completed the questionnaires in all three stages [7].

Notably, the results demonstrate that participants can continue to enact or maintain changes in various domains of food literacy. Participants continue to increase their frequency of plan and manage and selection behaviours, which indicates continuing practice at home and retaining knowledge and skills. The three-month follow-up questions may not accurately indicate the preparation domain changes—the questions regarding trying new recipes and changing recipes to be healthier may not be relevant to every participant as some may have already implemented these changes and are no longer actively trying new recipes or changing recipes to be healthier. Participants commonly reported cooking-related beneficial reasons for attending the program. Self-reported cooking skills improved significantly from the beginning of the program, which may support the idea that preparation changes had been implemented, but continued support and reminders were required to continue these behaviours.

Regarding dietary intake, the results decreased in the follow-up time period but did not return to their original level from the beginning of the program (an exception was sugar-sweetened drinks, which did not change throughout the program). Further research is required to understand the barriers to increasing healthy food intake when participants are reporting improving or sustaining food literacy behaviours. Many programs have documented increases in fruit and vegetable consumption; however, they may not have included measures of dietary behaviours or intake in the follow-up evaluation or included a follow-up evaluation at all [2,3]. The UK and Australian versions of the ‘Ministry of Food’ provide comparable examples of measuring dietary intake [7,10]. Within the eight-week Australian version, a 0.52 serve increase of vegetable consumption and a 0.4 serve increase of fruit consumption remained evident at the six-month follow-up evaluation. Despite the program’s aim to reduce the consumption of fast food and ready-made meals, there was only a statistically significant reduction in fast food consumption, not ready-made meals post-program, which did not change at follow-up.

Participants’ perceptions of their preparation and cooking skills are associated with the frequency of all food literacy behaviours. The preparation and cooking domain of food literacy appears to be the active ingredient for individuals using food literacy to improve their dietary intake. Fredericks et al. discussed the ten experiential features of culinary or cooking programs that instigate behavioural changes based on the experience of nutrition educators [26]. At the beginning of the FSA program, 68% (*n* = 1605) of participants rated themselves as being able to cook almost anything or cook a wide variety of meals; at the follow-up, participants improved the perception of their skills [20]. A food literacy program’s ability to build confidence and provide challenges, successes, peer support, and collaboration influences behavioural changes [27] and provides opportunities to practice new behaviours [28].

Food literacy is a complex set of knowledge, skills and behaviours. Therefore, it is a complex process to transform knowledge and skills into positive behaviours successfully, and this change requires further research. Food literacy programs tend to attempt to change cognitions, such as attitudes and apply constructs from social cognitive models, such as improving self-efficacy [29]. Applying these theoretical models utilises numerous strategies to change the determinants of behaviour. However, limitations are recognised in their predictive power for long-term behavioural change and unclear guidance regarding how to operationalise theoretical constructs while delivering curriculum [30]. Atkins and Michie developed a behaviour change taxonomy and identified the key drivers that support an individual’s motivation and capability and the opportunities that individuals have to practice or habituate new behaviours [31]. Identifying the participants that maintain or continue change will assist in understanding techniques to change food literacy behaviours to inform program content [31].

Further research is required to understand the cues for food literacy behaviour change and the reward received when making changes that assist in creating habits. Opportunities for specific participants to attend advanced programs or refresher programs may assist in maintaining and improving behaviour change. In addition, technology support applications and programs could provide reminders regarding using food literacy to improve fruit and vegetable intake and reduce fast food meal intake [32,33]. Participants who complete a food literacy program require support from multiple spheres of influence, including households, setting and the environment as outlined in the socio-ecological model for continued behavioural change [4]. Programs need to address behaviour change barriers, including addressing family taste preferences; environmental barriers, including the food prices, must be addressed with other interventions.

There are numerous limitations to acknowledge. First, the ideal follow-up or outcome evaluation time is unknown. At the three-month evaluation point, results may have missed potential changes in the preparation domain. Second, community-focused programs present challenges for evaluation because, unlike research trials, they do not contain a control group. It is also possible that participants may engage other strategies post-program to continue improvements; therefore, not all change can be attributed to the program. Third, the demographic characteristics of those completing the follow-up questionnaire are different. It may be the highly motivated participants that complete the questionnaire, which potentially introduces a retention bias. Fourth, the evaluation included several short self-reported dietary behaviour questions. It is necessary to deepen the understanding regarding the association between dietary behaviour change using comprehensive measures and food literacy behaviours, and the effect of barriers to using food literacy behaviours post-program to improve dietary intake. Lastly, all program evaluation data is reliant on self-reported responses and is, therefore, subject to social desirability bias, whereby participants respond in a socially acceptable way as opposed to reporting their actual behaviour. 

## 5. Conclusions

Participation in Food Sensations for Adults resulted in the more frequent use of plan and manage, selection, and (potentially) preparation skills in the longer term. Food literacy programs are effective; however, there is a need to continue assisting and supporting participants to implement their skills—particularly food preparation—to maintain changes. Further investigation strategies for supporting and maintaining behaviour change after program completion is required.

## Figures and Tables

**Table 1:** Demographic characteristics of Food Sensations for Adults (FSA) participants who did and did not complete the follow-up questionnaire.

Characteristic.	Responses	FSA Respondents: Completed End of Program and Follow-Up Questionnaires (%)	FSA Respondents: Completed End of Program Questionnaire Only (%)	*p*-Value
Sex (*n* = 544, 422)	Male	86 (15.8%)	122 (21.6%)	0.0131
	Female	458 (84.2%)	442 (78.4%)	
Age group (years) (*n* = 543, 566)	18–25y	44 (8.1%)	84 (14.8%)	0.0001
	26–35y	109 (20.1%)	144 (25.4%)	
	36–45y	140 (25.8%)	132 (23.3%)	
	46–55y	75 (13.8%)	78 (13.8%)	
	56–65y	78 (14.4%)	64 (11.3%)	
	66 and over	97 (17.9%)	64 (11.3%)	
Household composition (*n* = 543, 562)	Single person	99 (18.2%)	89 (15.8%)	<0.0001
	Partner	114 (21.0%)	93 (16.5%)	
	Single parent with child/children	51 (9.4%)	55 (9.8%)	
	Couple with children	213 (39.2%)	189 (33.6%)	
	Other: Family/Extended family/Shared/ Supported accommodation	66 (12.2%)	136 (24.2%)	
Education level (*n* = 543, 555)	Some secondary/ finished primary school	74 (13.6%)	113 (20.4%)	0.0002
	Finished high school	115 (21.2%)	147 (26.5%)	
	Certificate/Diploma/Trade	215 (39.6%)	167 (30.1%)	
	Bachelor or higher	139 (25.6%)	128 (23.1%)	
Employment status (*n* = 542, 555)	Full-time /self-employed	49 (9.0%)	59 (10.6%)	0.0002
	Part-time/ casual	130 (24.0%)	122 (22.0%)	
	Unemployed /unable to work	107 (19.7%)	159 (28.6%)	
	House duties/maternity leave/retired	256 (47.2%)	215 (38.7%)	
Socioeconomic Index ^1^ (*n* = 534, 537)	Low	238 (44.6%)	234 (43.6%)	0.1730
	Middle	134 (25.1%)	160 (29.8%)	
	High	162 (30.3%)	143 (26.6%)	
Born in Australia ^2^ (*n* = 534, 522)	Yes	293 (54.9%)	310 (59.4%)	0.1380
	No	241 (45.1%)	212 (40.6%)	
Identify as Aboriginal or Torres Strait Islander ^2^ (*n* = 527, 521)	Yes	18 (3.4%)	45 (8.6%)	0.0004
	No	509 (96.6%)	476 (91.4%)	

^1^ Socio-Economic Indexes for Areas (SEIFA) derived from postcode [24]. ^2^ Added in a later version of the questionnaire.

**Table 2:** Comparing end-of-program and follow-up questionnaire scores regarding the three food literacy behaviour domains and changes in self-reported dietary intake of fruit and vegetables.

	End-of-Program (Mean)	Follow-Up (Mean)	*p*-Value	95% CI of Difference—Lower	95% CI of Difference—Upper	Difference (End-of-Program Follow-Up) (%)
Food literacy behaviours						
Plan and Manage (*n* = 552)	9.99	10.29	<0.0001	–0.42	–0.18	3.04
Selection (*n* = 589)	3.77	4.04	<0.0001	–0.37	–0.17	7.16
Preparation (*n* = 576)	7.2	6.98	0.0002 *	0.11	0.34	–3.06
Dietary intake behaviours						
Serves of fruit (*n* = 590)	1.84	1.74	0.0186 **	0.02	0.17	–5.43
Serves of vegetables (*n* = 589)	3.01	2.77	<0.0001 ***	0.15	0.33	–7.97

* Preparation: follow-up—start difference: 9.52%, *p* < 0.0001; ** Serves of fruit: follow-up—start difference: 11.94%, *p* < 0.0001; *** Serves of vegetables: follow-up—start difference: 16.82%, *p* < 0.0001.

**Table 3:** Self-reported cooking skills comparison between the program’s beginning and follow-up questionnaires.

Self-Reported Cooking Skills (*n* = 540, *p* < 0.0001)					
		**Follow-Up** *n* (%) *			
		**Can cook almost anything**	**Can cook a wide variety of meals**	**Can cook basic food (e.g., meat and three vegetables)**	**Can only do basic heating/cannot or does not cook**
**Beginning-of-program** *n* (%) *	**Can cook almost anything**	85 (64.4%)	39 (29.5%)	8 (6.1%)	0
	**Can cook a wide variety of meals**	41 (16.9%)	182 (75.2%)	14 (5.8%)	5 (2.1%)
	**Can cook basic meat and three vegetables**	3 (2.2%)	59 (43.7%)	68 (50.4%)	5 (3.7%)
	**Can only do basic heating/cannot or does not cook**	3 (9.7%)	6 (19.4%)	14 (45.2%)	8 (25.8%)

* Percentages refer to the specific beginning-of-program population.

**Table 4:** Comparison between end-of-program and follow-up number of participants in low, moderate and high scoring groups for the three food literacy behaviour domains (a) plan and manage, (b) selection, and (c) preparation.

Plan and Manage (*n* = 552, *p* = 0.0015) *				
		**Follow-Up**		
		**Low**	**Moderate**	**High**
**End of Program**	**Low** **Moderate** **High**	26 (40.0%) 15 (8.6%) 8 (2.6%)	25 (38.5%) 73 (41.7%) 48 (15.4%)	14 (21.5%) 87 (49.7%) 256 (82.1%)
**Selection** (*n* = 589, *p* < 0.0001) *				
		**Follow-Up**		
		**Low**	**Moderate**	**High**
**End of Program**	**Low** **Moderate** **High**	20 (26.7%) 17 (9.7%) 11 (3.2%)	25 (33.3%)	30 (40.0%) 97 (55.4%) 279 (82.3%)
			61 (34.9%)	
			49 (14.5%)	
**Preparation** (*n* = 576, *p* = 0.0985)				
		**Follow-Up**		
		**Low**	**Moderate**	**High**
**End of Program**	**Low**	47 (56.0%)	23 (27.4%)	14 (16.7%)
	**Moderate**	36 (18.7%)	94 (48.7%)	63 (32.5%)
	**High**	21 (7.0%)	80 (26.8%)	198 (66.2%)

* Percentages refer to the specific beginning-of-program population (low, moderate, or high).

**Table 5:** Demographic and dietary behaviours associated with maintained or increased food literacy behaviour between end-of-program and follow-up questionnaires (those scoring high at the program’s end and on follow-up, or scoring moderate at the program’s end and scoring moderate or high on follow-up). ns: not statistically significant.

Characteristic	Responses	Plan and Manage (*n* = 441)	Selection (*n* = 459)	Preparation (*n* = 446)
**Socio-Economic Index ^1^**	Low	1	1	ns
	Moderate	1.30 (0.74–2.27) *p* = 0.3673	1.25 (0.74–2.11) *p* = 0.4004	
	High	2.06 (1.16–3.65) ***p* = 0.0138** ^2^	2.03 (1.17–3.5) ***p* = 0.0111**	
**Self-Described Cooking Skills**	Can cook almost anything	9.99 (3.83–26.06) ***p* < 0.0001**	2.33 (0.96–5.68) *p* = 0.0626	3.77 (1.54–9.26) ***p* = 0.0037**
	Can cook a wide variety of meals	10.01 (4.11–24.41) ***p* < 0.0001**	2.80 (1.2–6.5) ***p* = 0.0168**	2.56 (1.1–5.96) ***p* = 0.0285**
	Can cook basic food (e.g., meat and three vegetables)	3.48 (1.41–8.59) ***p* = 0.0067**	1.05 (0.44–2.47) *p* = 0.9142	1.08 (0.45–2.59) *p* = 0.8599
	Can only heat food/cannot or does not cook	1	1	1

^1^ Socio-Economic Indexes for Areas (SEIFA) derived from postcode [24]. ^2^ Bold indicates p-values considered statistically significant.

## Supplementary Materials

The following are available online at https://www.mdpi.com/1660-4601/17/12/4462/s1, S1: Food Sensations Start of Program Questions.
